# Harmful effects of carbamazepine on the postnatal development of the rat ventral prostate

**DOI:** 10.1186/1477-7827-10-22

**Published:** 2012-03-25

**Authors:** Samara U Oliva, Wellerson R Scarano, Fatima K Okada, Sandra M Miraglia

**Affiliations:** 1 Department of Morphology and Genetics, Developmental Biology Laboratory, Federal University of São Paulo (UNIFESP), São Paulo, SP, Brazil; 2 Department of Morphology, São Paulo State University (UNESP), Botucatu, SP, Brazil

**Keywords:** Carbamazepine, Ventral prostate, Mast cells, Stereology

## Abstract

**Background:**

Carbamazepine (CBZ) is a first-line antiepileptic drug (AED), although it is also used for the treatments of psychiatric disorders and neuropathic pain. The CBZ utilization has been associated with male reproductive damage, including hormonal alterations, sexual dysfunction and reduction of sperm quality. The wide and long-term use of the CBZ is a common schedule in children and adolescents and alters the testosterone level in adult rats and humans. The objective of this work was to evaluate the CBZ side effects on the ventral prostate of rats from pre-puberty to sexual maturation, since the prostate is an androgen-dependent organ.

**Methods:**

Twenty three day-old male albino Wistar rats received CBZ diluted in propylene glycol (20 mg/Kg/i.p via). The treatment lasted 20, 40 and 70 days, according to the different stages of the rat sexual maturation. At the end of each treatment period, ventral prostates were removed and histologically processed. The prostate sections were submitted to the histopathological, morphological and stereological analyses using image analysis system.

**Results:**

Reductions of the glandular epithelium, glandular lumen and fibromuscular stroma volume of the ventral prostate were observed in adult rats treated with CBZ since the weaning. Triggering and degranulation of mast cells were observed in the fibromuscular stroma of prepubertal and pubertal CBZ treated rats.

**Conclusions:**

The results suggest a direct effect of the CBZ on rat ventral prostate, evidenced by increase of mast cell and macrophage populations during pre-puberty and puberty causing a ventral prostate accentuated damage in the adult phase.

## Background

Reproductive endocrine disorders and sexual dysfunction are common in men with epilepsy [1]. In addition, these disorders have been frequently associated with long-term treatments with antiepileptic drugs (AED) [2-4]. CBZ is a first-line AED also utilized for treatment of psychiatric disorders such as bipolar affective disorder [5], alcohol withdrawal syndrome [6] and neuropathic pain [7].

Various clinical and experimental studies involving the effects of CBZ and other AED on male reproduction have been carried out. They have mainly shown hormonal changes and seminal alterations during adult phase, including reduced sperm motility, sperm morphological alterations and decrease in the sperm concentration [8-10]. However, studies have not investigated the occurrence of damage to the prostate in the phase of sexual maturation when the CBZ is chronically administered from the pre-pubertal phase.

The reproductive hormone status differs noticeably in humans, according to the phase considered, i.e., in childhood, adolescence and adulthood. Thus, endocrine changes due to long-term CBZ-treatments may cause a negative impact on pubertal development and fertility both in boys and young men [11]. Damage on the seminiferous epithelium, testicular interstitial oedema, reductions of testosterone levels and increase of estradiol levels were also observed in rats treated with CBZ since weaning [12].

The onset and maintenance of prostatic development are androgen-dependent phenomena [13]. However, in rodents, the prostatic development is also sensitive to other hormones such as estrogen and prolactin [14-16]. The prostate secretion is important for normal male reproductive function as well as sperm survival within the female reproductive tract during fertilization [14].

The responsive morphological alterations of the prostate to androgen deprivation caused by diabetes [17], chronic alcoholism [18], nicotine [19] and castration [16,20-22] have been shown. In addition, as the chronic administration of CBZ is a common schedule utilized for treatment of children and adolescents [11] and can provoke hormonal alterations as well as damage on fertility [8-12]. In addition, given that there are not studies investigating the extension of the prostatic damage, caused by CBZ chronic administration, during the sexual maturation, we assumed that this theme is an essential subject to be investigated. Thus, considering that the maturation of the hypothalamic-pituitary-gonadal (HPG) axis in pre-puberty and puberty is more susceptible to a cytotoxic agent [12,23,24] and also, taking into account that recently we observed estrogen and testosterone level alterations in CBZ-treated rats from the weaning [12], we proposed to evaluate the side effects of CBZ on the ventral prostate of rats in different phases of sexual development; for this goal, the CBZ was administered to rats from the pre-puberty until the adulthood and the histopathological, morphometric and stereological analyses of their prostate ventral lobe were carried out.

## Methods

### Animals

Thirty 23-day old male Wistar albino rats were used. The rats were housed in polypropylene cages (40 × 30 × 15 cm) (4 per cage) and had free access to water and commercial lab chow (Labina, Purina). The animals were maintained under controlled photoperiod (12 h light; 12 h dark), humidity (70%) and temperature (22°-23°C). The experimental protocol followed the ethical principles adopted in animal researches by the Brazilian College of Animal Experimentation. The schedule concerning animal care and treatment was approved by the Institutional Ethical Committee of São Paulo University.

### Experimental schedules

The animals were randomly distributed into six groups with 5 animals each. The rats were distributed into three control groups (C43, C63 and C93) and three CBZ-treated groups (CBZ43, CBZ63 and CBZ93). Treated rats received CBZ (C-8981, Sigma Chemical Co., St. Louis, MO; 99.5% purity) diluted in propylene glycol (20 mg/ml). The CBZ dose of 20 mg/kg, daily administered by intraperitoneal route (i.p.), corresponds to the usual anticonvulsive dose in humans which is effective in preventing kindled seizure in Wistar rats [25,26]. The treatment lasted 20, 40 and 70 days, according to the different stages of the rats' sexual maturation, i.e., adolescence (around 43 days; [12,27]), puberty (63 days; [28,29]) and young adult phase (93 days; [30]). Control animals received only propylene glycol (vehicle of CBZ). Body weights of control and experimental rats were obtained on alternate days.

### Histological procedures

At the end of the different treatment periods, the rats were anesthetized with sodic thiopental (1 ml/kg, Thiopentax, Cristália, Lote 06053324). Heparin (1 ml/Kg) was administered 15 minutes before the euthanasia with anesthetic superdosage (sodic thiopental 3 mg/kg, i.p.), by i.p. route. Blood of the rats was collected from the inferior cava vein and the plasma separated and stored at -20°C for further hormonal analyses. The ventral prostate was removed and weighed. The ventral prostate relative weight (mg of ventral prostate weight/100 g of body weight) was also calculated. The volume of the ventral prostate (Vt) was obtained according to DeKlerk & Coffey's methodology [31], which assumes that the specific gravity of the prostate is 1.0 and thus, 1.0 mg fresh prostate has an approximate volume of 1.0 mm^3^.

The ventral prostates were adequately fixed by immersion in Bouin's liquid for 48 hours. Prostatic fragments were Paraplast Plus-embedded (P-3683, Sigma Chemical Co., St. Louis, MO, lote 41K0101); five micrometers-thick cross sections were obtained and stained using Mallory Tricromic, for subsequent morphometric and stereological studies. In addition, other five sections were obtained and exposed to toluidine blue staining method and to Perl's solution labeling for respective stereological evaluation and the obtainment of the mast cells and macrophage numerical densities.

Fragments were also embedded in glycol methacrylate resin (Leica Historesin embedding kit); two micrometer-thick longitudinal sections were obtained and submitted to the Hematoxilin and Eosin (HE) staining method for histopathological analysis under light microscope.

### Macrophage labeling

For macrophage labeling, paraplast-embedding tissue sections were immersed in Perl's solution containing 2% potassium ferrocyanide and 2% HCl at a 1:1 concentration for 30 min [32]. After PBS rinses and 30 min incubation in 3% hydrogen peroxide/PBS, iron was visualized using 3,3-diaminobenzidine (DAB; Vector) for 30 min at room temperature in the dark [33].

### Morphometric and stereological evaluation

Samples of the ventral prostate were obtained according to the orientator to produce an isotropic uniform random (IUR) sample [34,35]. Each ventral prostate sample was divided into parallel slabs of roughly equal length and each slab was cut into parallel vertical slices. The angle at which the first slab was cut was chosen at random and other slabs were cut in similar fashion after systematically rotating on the horizontal plane, providing an isotropic uniform random section plane. The IUR sections are more random than vertical sections and therefore can be used as vertical sections with cycloids for estimation of length and surface [36].

The morphometric and stereological analyses of the compartments of the prostate ventral lobe were carried out. The volume densities (**Vv**) of the lumen **[Vv(lu)]**, glandular epithelium or acinar parenchyma **[Vv(ep)] **and fibromuscular stroma **[Vv(st)] **were calculated using a Leica QWin (Cambridge, England) image analysis system. Twenty two fields were systematically randomly sampled and the images were captured using a digital camera connected to a light microscope. Examination of isotropic uniform random (IUR) sections of the ventral prostate revealed that the glandular tubules were randomly placed [20]. The Delesse Principle states that the planimetric fraction of a section, which is occupied by sections of a specified component, corresponds to the fraction of the tissue volume occupied by the same component [37]. For that reason, according to the basic principle of stereology, the area density of the profiles (**A_A_**), which measures the relative occupation of test-area for the area of the images of the structure under evaluation, is comparable to the volume density (**Vv**), i.e., the density of profiles in relation to the space; consequently, **Vv **and **A_A _**have similar interpretation [37-40]. Thus, from the captured images, the ventral prostate lumen, glandular epithelium and fibromuscular stroma areas were delineated, utilizing the image analysis system. Then, based on the principles of stereology, each specific volume density (**Vv**) was respectively obtained by the ratio of the delineated area of the ventral prostate (lumen, glandular epithelium or fibromuscular stroma compartment) to the total area of the prostate component analyzed. In addition, the volumes (**V**) of the lumen (**V***lu*), glandular epithelium (**V***ep*) and fibromuscular stroma (**V***st*) of the ventral prostate were estimated by multiplying each respective volume density (in percentage) by the prostate total volume (in "Histological procedures" item) and dividing the result by 100 [29].

The determination of length density (Lv) of the glandular tubule (Lv*gt*) and surface density of the glandular epithelium tubule (Sv*gt*) were also performed [21]. To measure the length density of the glandular tubules (Lv*gt*), we firstly calculated the profile density of the structure evaluated (Q_A_), i.e., the number of glandular tubule profiles per area unit was counted. For this purpose, an amount of images of these structures (sections of glandular tubules) was estimated in a certain test-area defined by an unbiased counting frame with inclusion (right and upper) and exclusion (left and lower) borders; thus, a reliable square test-area was superimposed on the microscopic images IUR ventral prostate section captured and shown by the monitor screen of the image analysis system. Twenty two fields were systematically randomly sampled. Following, the number of glandular tubule profiles inside the total test area was counted (Q_A_). All structures falling on the two set forbidden lines were not counted to avoid overestimation. Subsequently, the glandular tubule length density (Lv*gt*) was obtained by the formula: Lv*gt *= 2 * Q_A _[35]. The total length (L) of glandular tubules (L*gt*) was also calculated using the formula: L*gt *= Lv*gt *x Vt, where Lv*gt *corresponds to the glandular tubule length density [35] and Vt is the ventral prostate total volume obtained as formerly referred. The total area analyzed was obtained by multiplying the area of the test grid (1874.75 mm^2^) by the number of fields analyzed (23 fields) utilizing ×20 objective lens.

The surface density of the glandular epithelium Sv*ep *was obtained by counting the number of intercepts of the cycloid test lines with luminal border of the glandular epithelium using a cycloid test grid [37]. Then, the glandular epithelium surface (S*ep*) was calculated by multiplying Sv*ep *by the ventral prostate total volume (Vt) and dividing this result by 100 [20].

### Numerical densities of mast cells and macrophages

Twenty two fields were systematically randomly sampled and the images were captured using a computer assisted image analysis system (Leica-Q550IW; Cambridge-England) coupled to a light microscope. Both macrophage (Perl's solution labeling) and mast cells (toluidine blue staining) counts were attained by Optical Disector method. Considering that the numerical density (Nv) is expressed by units of volume and corresponds to the ratio of total number of scored cells to total volume of tissue examined (V), the numbers of macrophage and mast cells per mm^3 ^of fibromuscular stroma were obtained. The volume of the fibromuscular stroma (v) was estimated by multiplying the respective areas delineated (in mm^2^) by thickness cross sections (in mm). Thus, the numerical density (Nv) is expressed by units of volume and corresponds to the ratio between the total number of scored macrophage/mast cells and volume of fibromuscular stroma examined. Counts of macrophage and mast cells were conducted. To avoid overestimation, we scrutinized the testicular tissue by focusing only on one chosen plane (the look-up plane; [41]); thus, only the tissue viewed on this plane was submitted to stereological analysis [42].

### Immunohistochemistry (IHC)

AR (SC-816, rabbit polyclonal IgG, epitope mapping at the N-terminus of AR, Santa Cruz Biotechnology, USA) primary antibody was used for immunohistochemistry (IHC). The IHC reaction was performed using the avidin-biotin complex (ABC) kit (Santa Cruz Biotechnology, CA, USA). For the immunohistochemical technique, the ventral prostate fragments were previous adequately fixed by immersion in Bouin's liquid and processed for paraplast-plus embedding (P-3683; Sigma Chemical Co., lote 41K0101). The sections (5 μm) were dewaxed and then rehydrated in alcohol graded solution and distilled water. Antigenic retrieval was realized in citrate buffer at high temperature (100°C) for 45 minutes. Endogenous peroxidase activity was blocked with 0.3% hydrogen peroxide in methanol for 45 min, followed by a quick rinse in distilled water and phosphate-buffered saline (PBS), and then incubated in 1% bovine serum in PBS for 1 h, to block non-specific binding. Sections were incubated with the primary antibody at 4°C overnight. The slides were then incubated with biotinylated secondary antibody at 37°C followed by peroxidase-conjugated avidin-biotin complexes and diaminobenzidine (DAB). The sections were then counterstained with Harris's hematoxylin. For negative control, the primary antibody was replaced with the corresponding normal isotype serum.

### AR semi-quantitative analysis

At least five histological sections were analyzed per group and AR positive nuclei were randomly selected by section (100 nuclei/group). The images of the selected nuclei were cut in a regular and constant rectangular form (always in central part of the nucleus, in view of the homogeneity of nuclear staining/reactivity for AR) and those "rectangles" were submitted to optical densitometry analysis by Scion Image for Windows Software^®^. The integrated optical density (IOD) methodological proceedings were based on physical principles of quantification performed [39].

### Hormonal levels

Testosterone and estradiol plasmatic levels were determined by radioimmunoassay method using Coat-A-Count Total Testosterone kit and Coat-A-Count Total Estradiol kit (Diagnostic Products Corp., LA, CA, USA). The sensitivity of all Kits utilized was 0.01 ng/mL.

### Statistical analysis

Data obtained from the treated and control groups were compared using two-way ANOVA; post hoc "t" parametric test (Student test) or the non-parametric Mann-Whitney tests were utilized. The differences were considered statistically significant when *p *≤ 0.05.

## Results

### Morphometric and stereological evaluation

Treatment with CBZ caused a significant decrease in the ventral prostate absolute weight of the CBZ93 rats in comparison to the respective C93 control rats. In addition, prostatic volume and total length of glandular tubules also significantly diminished in the CBZ93 rats. On the other hand, the absolute and relative weights of the ventral prostate as well as the prostatic volume and length of rats from the other CBZ-treated groups (CBZ43 and CBZ63) did not show significant statistical differences in comparison to their control groups (Table 1).

**Table 1:** Prostatic morphometric and stereological evaluation

Groups	Absolute prostatic weight (mg)	Relative prostatic weight (mg/100 g b.w.)	Prostatic volume (mm^3^)	Lv*gt *(1/mm^2^)	L*gt *(mm)
C43	110.50 ± 26.25	66.11 ± 14.38	110.50 ± 26.25	27.10 ± 5.61	2,97 ± 0.84

CBZ43	87.60 ± 8.56	60.08 ± 3.83	87.60 ± 8.56	32.70 ± 6.56	2.89 ± 0.77

C63	245.72 ± 21.64	83.80 ± 7.95	245.72 ± 21.64	23.10 ± 2.36	5.71 ± 1.05

CBZ63	231.00 ± 9.17	81.48 ± 5.91	231.00 ± 9.17	20.40 ± 5.99	4.70 ± 1.35

C93	423.08 ± 74.44	12.24 ± 0.32	423.08 ± 74.44	19.10 ± 2.75	8.07 ± 1.79

CBZ93	260.24 ± 46.80*	12.01 ± 0.67	260.24 ± 46.80*	21.50 ± 4.03	5.56 ± 1.47*

Data are reported as mean ± SD.

Differences statistically significant: *p ≤ 0.05

Prostate absolute and relative weights, prostate volume, length density (Lv*gt*) and length (L*gt*) of the prostate glandular tubules in the control and CBZ-treated groups

**Figure 1:**
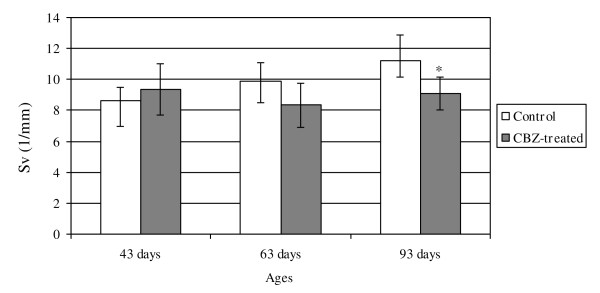
**Surface density (Sv) of the glandular tubule epithelium of the (Sv*gt*) in the prostate of control and CBZ-treated rats**. Surface density (Sv) of the glandular tubule epithelium of the (Sv*gt*) in the prostate of control and CBZ-treated rats. Mean ± SD; n = 5. Statistically significant differences: *p ≤ 0.05.

**Figure 2:**
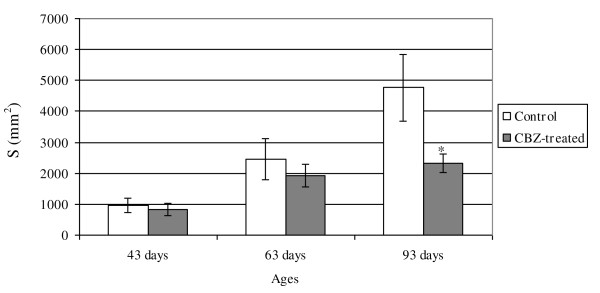
**Surface (S) of the epithelium of the prostatic glandular tubules (S*gt*) in the control and CBZ-treated groups**. Surface (S) of the epithelium of the prostatic glandular tubules (S*gt*) in the control and CBZ-treated groups. Mean ± SD; n = 5. Statistically significant differences: *p ≤ 0.05.

Reductions of the volume of the ventral prostate glandular lumen, and of glandular epithelium and fibromuscular stroma were also observed in the CBZ93 group comparatively to their respective control rats (Table 2). Moreover, a decrease in the surface density (Sv) and surface (S) of the epithelium of the glandular tubules were also noted in the CBZ93 group (Figures 1 and 2).

**Table 2:** Volume density (Vv) and total volume (V)

Stereological and morphometric parameters	Groups	
	
	C93	CBZ93
Vv glandular epithelium (%)	39.14 ± 5.90	38.69 ± 4.68

Vv glandular lumen (%)	43.31 ± 9.40	41.83 ± 6.67

Vv fibromuscular stroma (%)	17.55 ± 5.34	19.49 ± 2.79

V glandular tubule epithelium (mm^3^)	162.47 ± 14.99	101.11 ± 22.88*

V glandular lumen (mm^3^)	188.69 ± 70.08	108.38 ± 22.26*

V fibromuscular stroma (mm^3^)	71.92 ± 13.46	50.74 ± 11.86*

Data are reported as mean ± SD; n = 5. Statistically significant differences: *p ≤ 0.05

Volume density (Vv) and total volume (V) of the prostatic glandular epithelium, glandular lumen and fibromuscular stroma in the C93 control and CBZ93 groups

The volume and volume density of the ventral prostate lumen, epithelium of the glandular tubules and stroma compartments of the CBZ63 and CBZ43 did not show significant alterations in comparison with the control rats of the same age (data not shown).

### Histological analysis and numerical densities of mast cells and macrophages

**Figure 3:**
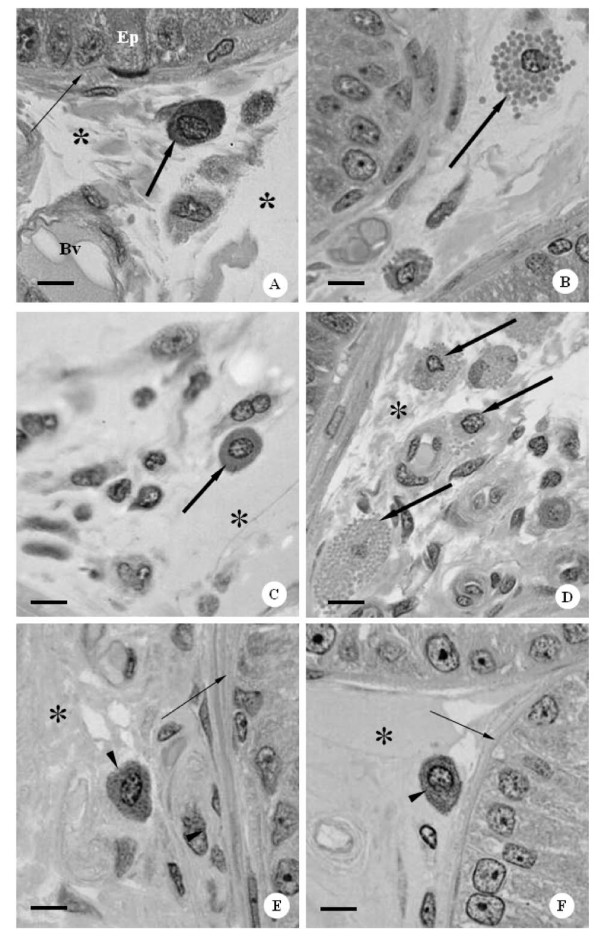
**Photomicrographs of ventral prostate sections of control and CBZ treated rats at different ages**. HE-method. **A**: Part of ventral prostate section of 43-day old control rat, showing glandular epithelium with tall columnar secretory epithelial cells (Ep), smooth muscle layers (thin arrow) and connective tissue (asterisks). Note the presence of no activated mast cell in the fibromuscular stroma (thick arrow). Bv: blood vessel. Bar: 9 μm. **B**: Part of ventral prostate section of CBZ43 rat. Note the mast cell in the fibromuscular stroma with visible cytoplasmatic granules (thick arrow). Bar: 9 μm. **C**: Part of ventral prostate section of a 63-day old control rat, showing detail of the fibromuscular stroma (asterisk) containing mast cell (thick arrow) with no conspicuous cytoplasmatic granules. Bar: 9 μm. **D**: Ventral prostate section of the CBZ63 rat. Note the various mast cells in process of degranulation (thick arrows) disposed in the fibromuscular stroma (asterisk). Bar: 10 μm. **E **and **F**: Ventral prostate sections of C93 and CBZ93 rats. Note the smooth muscle layers (thin arrows) and the connective tissue (asterisks) containing the mast cells with fine and few evident cytoplasmatic granules (head arrows). Bar: 7 μm and 7 μm, respectively.

**Figure 4:**
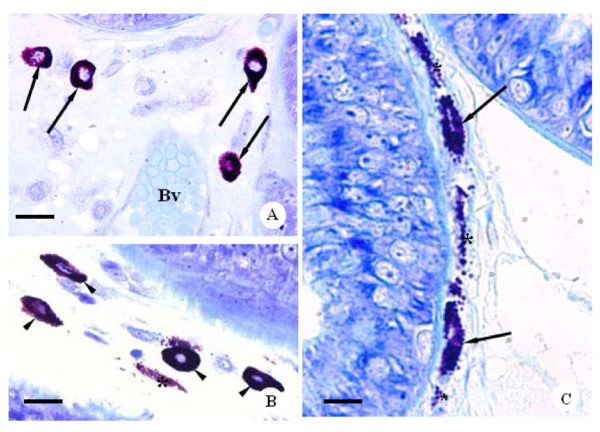
**Photomicrographs of ventral prostate sections of CBZ-treated rats at different ages**. Toluidine blue method. **A**: Part of ventral prostate sections of the CBZ93 rat showing the fibromuscular stroma with mast cells (arrows) around blood vessel (Bv). Bar: 20 μm. **B **and **C**: Ventral prostate sections of the CBZ63 rats. Note mast cells with fine cytoplasmatic granules (4b, head arrows), mast cells in degranulation process (4c, arrows) and cytoplasmatic granules (asterisks) distributed into the fibromuscular stroma. Bar: 18 μm and 11 μm, respectively.

**Figure 5:**
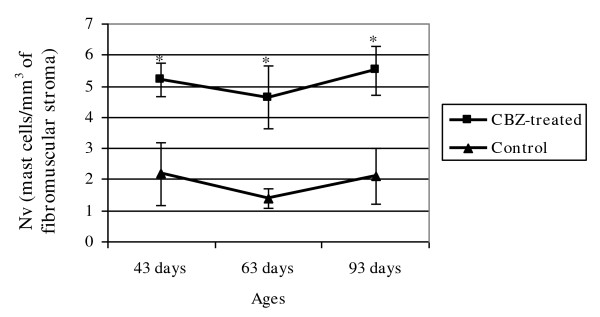
**Numerical density of the mast cells in the prostatic fibromuscular stroma in the control and CBZ-treated groups**. Mean ± SD; n = 5. Statistically significant differences: *p ≤ 0.05.

The sections of the rat ventral prostate of all control and CBZ-treated groups showed acini surrounded by a fibromuscular stroma, which frequently showed secretion within the lumen. In all groups, the mucosa of the ventral prostate was typically folded showing tall columnar secretory epithelial cells (Figures 3A, E). In the fibromuscular stroma, layers of smooth muscle fibers were observed involving the acini; outer and extensive connective tissue surrounded these structures (Figures 3A-F). Mast cells were found in the stroma of the control (Figures 3A, C, E) and CBZ-treated animals (Figures 3B, D, F and 4A-C). However, cytoplasm of mast cells showed accentuated granules in CBZ43 (Figure 3B) and CBZ63 groups (Figures 3D and 4B, C). Frequently, the granules appeared to be releasing into the connective tissue, suggesting an activation of the mast cells (Figure 3D and 4C). Nevertheless, the CBZ93 rats (Figure 3F) showed mast cells with similar morphological characteristics when compared to those of the C93 control group (Figure 3E). Moreover, the numerical density of mast cells was significantly higher in CBZ63 and CBZ93 groups in comparison to corresponding controls groups (Figure 5).

**Figure 6:**
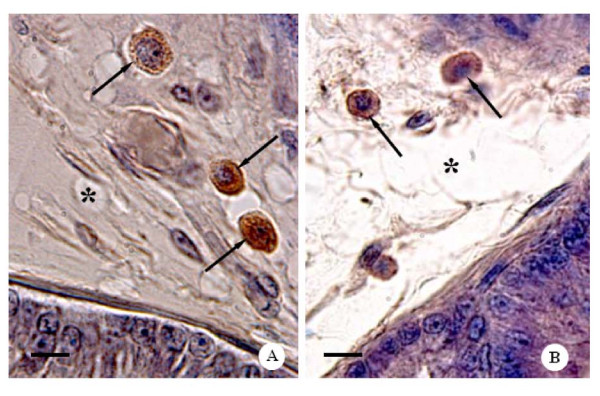
**Photomicrographs of ventral prostate sections of CBZ43 and CBZ63 rats, respectively**. **A **and **B**: Observe the positive staining macrophages (arrows) disposed in the fibromuscular stroma (asterisks). Perl's solution. Bar: 11 μm and 13 μm, respectively.

**Figure 7:**
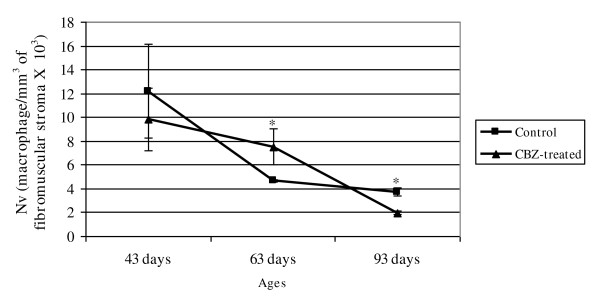
**Numerical density (Nv) of the macrophage on prostatic stroma fibromuscular in the control and CBZ-treated groups**. Mean ± SD; n = 5. Statistically significant differences: *p ≤ 0.05.

Macrophages were also located in the fibromuscular stroma of the control and CBZ-treated animals with 43, 63 and 93 days (Figures 6A, B). However, although the macrophage numerical density of the CZB63 group has shown a significant increase in comparison to the C63 control group, an evident reduction of this parameter occurred in the 93-day old CBZ-treated rats in relation to the C93 control rats (Figure 7).

### AR immunohistochemistry

**Figure 8:**
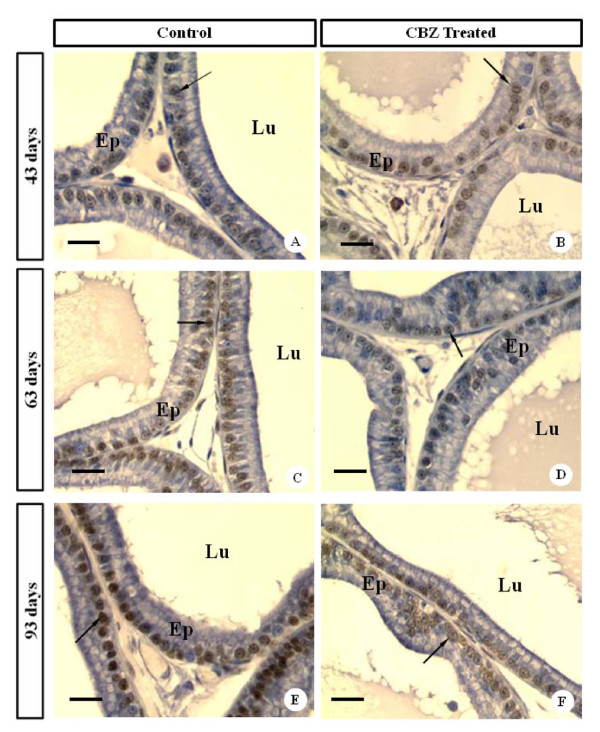
**Photomicrographs of ventral prostate sections of control and CBZ- treated rats at different ages submitted to AR IHC**. Brown stain signifies AR positive reactivity. Observe immuno-stainable AR located in the nuclei of cells in the prostate epithelium (arrows); AR immuno-localization showed decrease in staining intensity in 93- day old CBZ-treated rats when compared with their control rats. Ep: glandular epithelium; Lu: glandular lumen. Bars: 15 μm.

**Figure 9:**
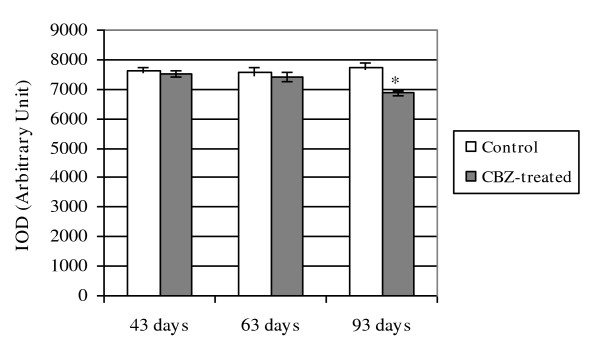
**Semi-quantitative analysis (IOD integrated optical density) of the nuclear epithelial cells AR imunoreactivity from ventral prostate in control and CBZ-treated groups**. Mean ± SD. Statistically significant differences: *p ≤ 0.05.

The ventral prostate AR immunoreactivity presented the same pattern of staining in the following control and treated groups: C43 and CBZ43; C63 and CBZ63 (Figures 8A-D). However, AR immunoreactivity was diminished in the CBZ93 group in relation to C93 control group (Figures 8E, F). The optical density of nuclear reactivity confirmed the qualitative analysis and showed decrease in the AR reactivity in the CBZ93 group when compared to the C93 control group (Figure 9).

### Hormonal levels

Plasma testosterone levels were also significantly lower in CBZ63 and CBZ93 rats in comparison with the C63 and C93 control rats (Table 3). Nonetheless, the 93-day-old CBZ-treated rats showed a significant increase in the estradiol plasma levels in comparison with the C93 control group, whereas there was not significant alterations in this hormone level in the 43-day-old and 63-day old CBZ-treated rats, in comparison with their respective control rats (Table 3).

**Table 3:** Hormonal plasma levels

Groups	Testosterone levels (ng/dL)	Estradiol levels (ng/dL)
C43	47.74 ± 8.06	17.19 ± 4.43

CBZ43	41.04 ± 6.9	24.45 ± 5.91

C63	262.64 ± 29.31	13.03 ± 1.39

CBZ63	115.11 ± 35.77*	14.03 ± 2.64

C93	373.99 ± 73.35	12.04 ± 2.32

CBZ93	267.15 ± 70.07*	18.74 ± 2.14*

Data are reported as mean ± SD.

Statistically significant differences: *p ≤ 0.05

Testosterone and estradiol plasma levels in controls (C43, C63 and C93) and CBZ-treated rats (CBZ43, CBZ63 and CBZ93)

## Discussion

In the prostate, death and cell proliferation are hormone regulated events. During the periods of normal growth of prostate, androgens are essential for stroma and epithelial cell differentiation throughout branching morphogenesis and ductal canalization [13,40]. At the age of sexual maturity, the secretory activity of the epithelium and the differentiation of smooth muscle are also maintained by androgens [40]. In the prostate, luminal and basal epithelium as well as stroma and smooth muscle cells express ARs at sexual development and hence are capable of mediating androgen's actions [43]. Thus, in response to androgens, cells of the prostate interact in a autocrine-paracrine way, influencing various aspects of the growth of this gland in normal and diseased states [13]. In fact, differential responsiveness of the prostate to androgens has been observed in rat at different phases of the sexual development [44,45]. On the other hand, CBZ treatment can cause alterations in the weight of seminal vesicle and prostate gland which are age-dependent; this probably occurs due to the higher responsiveness of the prostate to testosterone during the pre-puberty and adulthood than during the puberty [45,46]. During the pubertal period, testosterone clearance may be increase and this could account for the apparent reduction in prostate sensitivity during the pubertal period. It is possible that the alterations in prostate response reported are inherent to the tissue themselves and are dependent on their own spontaneous maturation independent of other hormonal events [47].

Puberty in the male rat is a complex process that involves maturational changes in the hypothalamus, pituitary, testes, and secondary sexual organs and in their interrelationships. During the course of sexual maturation, the negative feedback control systems for the gonadotropins become less responsive to testosterone while the testes become more responsive to LH. In the immature rat, testosterone can potentiate the effect of GnRH on pituitary LH release; this response is lost with sexual maturation. The responsiveness of the prostate to testosterone is also altered with age due proportion of testosterone and androstendione secreted by the testes. Experiments designed to prevent or mimic the transition in testicular steroid secretion suggest that it may be a critical component of sexual maturation in the male rat. An increase of androstendione appears to be capable of delaying the maturation of the LH negative feedback system, the prostate gland, and the GnRH self-priming effect [46]. Moreover, the metabolism of testosterone and/or alterations androgen receptor activity could be responsible for these sensibility differences [46]. In addition, estrogens also play a physiologic role in the prostate development with regard to programming stromal cells and directing early morphogenic events [48].

The significant reductions of ventral prostate weight and volume observed in CBZ93 rats probably occurred due to the CBZ-induced hormonal alteration. These data are in accordance with our previous study [12]; in this former study, a decrease in testosterone plasma levels and an increase in the estrogen level were respectively observed in 63 and 93 day-old rats which were also CBZ-treated from the weaning. In addition, the decrease in the weight of androgen-dependent organs as the ventral prostate is consistent with the decrease in serum levels of testosterone [13,49]. Besides, both androgen and estrogen regulate AR at the level of mRNA and protein. Then, abnormalities in the AR signaling pathway have also been linked to male reproductive alterations [50,51]. Moreover, post-transcriptional regulation through stability of the mRNA product is believed to be a major mechanism of androgens' effects [52]. In the current work, the CBZ treatment caused decrease in the AR reactivity of the 93-day old rat prostate; this event was confirmed by the optical density of nuclear reactivity. Prins and Birch [50] observed that neonatal chronic estrogen exposure resulted in an immediate and sustained decrease in AR protein levels in the developing and adult rat ventral prostate that in turn led to its abnormal growth and decreased secretory capacity.

In this present study, an increase of the estrogen plasma levels during puberty phase was observed in CBZ-treated rats. In addition, the estrogen plasma levels remained high in the adulthood and this phenomenon probably corroborated the reduction of the morphometric and stereological parameters evaluated. In fact, a decrease in the glandular acini length as well as in the glandular lumen, glandular epithelium and fibromuscular stroma volumes were observed in the CBZ93 group. These phenomena resulted in a reduction of the ventral prostate weight and volume in rats of this group. Additionally, we suggested that the reduction of glandular epithelium surface was a consequence of the diminution of the glandular epithelium volume. Thus, although CBZ chronic treatment since weaning has induced significant prostatic alterations observed in adult rats, the harm was started from the pre-puberty phase.

Similar to androgens, circulating levels of estradiol are high during the fetal and early neonatal life in both humans and rodent models [53] and there is compelling evidence that the developing prostate gland is particularly sensitive to these estrogens. Although the natural role for estrogens during prostatic development is unclear, it has been proposed that excessive estrogenization during prostatic development may contribute to the high incidence of benign prostatic hyperplasia and prostatic carcinoma [54]. Following neonatal exposure to high-dose estradiol, both epithelial and stromal cell proliferation and differentiation are markedly disturbed leading to defects that persist throughout the lifespan of the animal [55,56]. Neonatal estrogen exposure interrupts intercellular communication and blocks certain epithelial cells within the rat prostate from entering a normal differentiation pathway, beyond that the activational response to androgens during adulthood is permanently blunted in estrogenized rats [57] and this effect is mediated, in part, through an immediate and permanent reduction in prostatic AR expression [55,58-60]. Furthermore, the temporal expression patterns and quantitative levels of several other members of the steroid receptor superfamily are deregulated by early exposure to high doses of estradiol [15].

Besides, the possible direct effects of CBZ should be also considered as this drug is highly lipid-soluble [8,61]. Furthermore, some drugs alter the prostatic stroma-epithelial communication and this occurrence may affect the expression or distribution of growth factors, steroid hormones, and their respective receptors [13]. These phenomena corroborate the possible occurrence of a specific effect of the CBZ on ventral prostate, independently from the hormonal testosterone and estradiol level alterations caused by this drug.

Although structural alterations of the prostatic tissue have not been noted during the histopathological analysis, we could observe the presence of degranulated mast cells in fibromuscular stroma, both in the CBZ43 and CBZ63 rats.

There are mast cells in almost all organic systems, including lungs, skin, heart, and gastrointestinal tract. Depending on the organic system, differentiation state, stage of maturation as well as pathological conditions, mast cells can be heterogeneous in terms of their phenotype, functional properties, and local distribution [62,63]. In the ventral prostate, a quantitative analysis revealed a correlation between the numerical density of mast cells and the age of the rat, since their frequency increases during pre-puberty, remaining constant in the puberty and adult phases [64]. Mast cells participate in inflammatory reactions, angiogenesis, extracellular matrix reabsorption [65], fibrosis and tissue reconstruction [66]. Activated mast cells contain several inflammatory mediators, such as histamine, serotonin, cytokines, leukotrienos, prostaglandins, chemotatic substances, platelet-activing factor and potent vasodilators molecules [62]. These mastocitary products may be activated by different stimuli such as chemical substances, drugs, free radicals, estradiol and radiation [67]. Tobacco smoke, for example, can directly activate mast cells and to act releasing mediators such as histamine and tryptase, causing degranulation of mast cells [68,69].

The hormonal status may also induce activation and degranulation of mast cells. Estrogens can induce rat prostate inflammation [70] and then, mast cells can undergo degranulation [71]. Many experimental studies showed that steroid sexual hormones can influence the immune response and allergy development, in which estrogen is able to enhance humoral immune response and antibody synthesis, whereas androgen seems to exhibit inhibitory effects [72-74]. Therefore, the occurrence of activated and degranulated mast cells located in the fibromuscular stroma, of the CBZ63 group might be caused by alterations of the testosterone and estradiol levels.

Since changes of the testosterone or estradiol plasma levels this age were not at 43 days of age, the presence of activated and degranulated mast cells in the prostatic stroma of CBZ43 rats cannot be only explained based on hormonal alterations. Thus, in the our experiment, the frequent observation of activated and degranulated mast cells located in the fibromuscular stroma of the CBZ43 and CBZ63 groups again suggests that a direct effect of the CBZ on ventral prostate may have occurred, inducing possible transitory inflammatory process. In fact, mast cells granules containing preformed tumor necrosis factor α and releasing this cytokine from mast cells is important for the initiation of an inflammatory response [75].

Some experiments indicate that mast cell infiltration can enhance carcinogenesis [76,77]. Mast cells contribute to the development of skin cancer in K14-HPV16 transgenic mouse by proteases releasing, such as tryptase and chymase and stimulating angiogenesis [78].

In addition, the presence of degranulated mast cells in the prostatic stroma of CBZ63 rats was followed by increase of the macrophages number. This increase of macrophages might partly be due to the cytokines released from infiltrating mast cells. Macrophages have shown clearly to aid in both the initiation and progression of experimental cancers [79]. Similar increases in mast cells and macrophages were observed in rats exposure to 2-amino-1-methyl-6-phenylimidazo[4,5-b]pyridine (PhIP), a heterocyclic amine in cooked meat at high temperatures [80]; then, the inflammatory response to PhIP may help explain the tissue-specific and prostate lobe-specific carcinogenesis in the rat prostate induced by a long term dietary carcinogen [81]. Moreover, after castration, an increase of the mast cells can be observed in the rat prostate; besides, the number of ED-1-immunostained macrophages was also markedly increased in the epithelium and in the stroma of the rat ventral prostate [82].

In the present study, stereological and histomorphometric analyses revealed that CBZ chronic treatment since weaning causes, in the adult phase, decreases in: the volume of prostatic components, glandular epithelium surface and acini lenght. As a result, these alterations may have probably provoked reductions of the ventral prostate weight and volume in response to the CBZ direct effect. Besides, testosterone and estradiol plasma level alterations caused by CBZ can also indirectly act on the ventral prostate, influencing its maintenance and development. In addition, the presence of activated mast cells during pre-puberty and puberty phases may indicate a possible transitory inflammatory process, suggesting direct effect of the CBZ on ventral prostate.

In addition, we must remember that inflammatory reactions of the male genitourinary tract can affect male infertility in different degrees. In fact, high levels of cytokines secreted by mast cells can play a role in the decrease of the sperm function and sperm-egg interaction and this phenomenon have been widely neglected so far. Moreover, cytokines does not act only as key mediators of inflammation, but may also play important roles in the carcinogenesis and cancer progression [83,84]. Moreover, mast cells have been used as markers for risk stratification in invasive breast cancers [85,86]; then, it is possible that they can also function as cellular marker in other neoplasm, such as prostate tumor.

## Conclusions

Thus, particular attention must be drawn to the exposure to environmental contaminants and therapeutic drugs which can cause hormonal imbalances, injury and chronic inflammation of the prostate, leading to successive infertility and prostate cancer.

## Competing interests

The authors declare that they have no competing interests.

## Authors' contributions

SUO performed histological procedures, morphometric and stereological evaluation, histological analysis numerical, densities of mast cells and macrophages and hormonal levels determination. Immunohistochemistry and AR semi-quantitative analysis were carried out by WRS. SUO and WRS performed statistical analysis of the data. SUO, FKO and SMM conceived the study and drafted the manuscript. All authors read and approved the final manuscript.
